# Editorial: New horizons in the management of patients with atrial fibrillation: Interactions with the gastrointestinal system

**DOI:** 10.3389/fcvm.2022.1042728

**Published:** 2022-10-17

**Authors:** José Miguel Rivera-Caravaca, Gregory Y. H. Lip, Vanessa Roldán, Francisco Marín

**Affiliations:** ^1^Faculty of Nursing, Instituto Murciano de Investigación Biosanitaria (IMIB-Arrixaca), Centro de Investigación Biomédica en Red Enfermedades Cardiovasculares (CIBERCV), University of Murcia, Murcia, Spain; ^2^Liverpool Centre for Cardiovascular Science at University of Liverpool, Liverpool Heart and Chest Hospital, Liverpool John Moores University, Liverpool, United Kingdom; ^3^Department of Clinical Medicine, Aalborg University, Aalborg, Denmark; ^4^Department of Hematology and Clinical Oncology, Hospital General Universitario Morales Meseguer, Instituto Murciano de Investigación Biosanitaria (IMIB-Arrixaca), University of Murcia, Murcia, Spain; ^5^Department of Cardiology, Hospital Clínico Universitario Virgen de la Arrixaca, Instituto Murciano de Investigación Biosanitaria (IMIB-Arrixaca), CIBERCV, University of Murcia, Murcia, Spain

**Keywords:** atrial fibrillation, gastrointestinal system, oral anticoagulants, stroke, bleeding

The management of atrial fibrillation (AF), the most common cardiac arrhythmia, has evolved over the last years to be more multidisciplinary, comprehensive and integrated care based. This holistic view of the patient with AF is summed up as Atrial fibrillation Better Care (ABC) pathway (1), which is now recommended in many international guidelines (2–5).

This Research Topic of Frontiers in Cardiovascular Medicine includes three original articles, a mini-review and a case study which aim to shed light on some controversial and scarcely investigated topics in relation to the interaction of AF and the gastrointestinal system, and to explore novel approaches that should be considered for stroke prevention and risk-factor optimization.

Menichelli et al. investigated the relationship between circulating lipopolysaccharides (LPS) and antioxidant status in AF patients from the prospective, observational ATHERO-AF cohort. The authors measured gut-derived LPS and glutathione peroxidase 3 (GPx3), the blood isoform of GPx involved in the detoxification of superoxide anion. In the multivariable Cox regression analysis, the group of patients with serum LPS levels above median and serum GPx3 levels below median, had and independently higher risk of cardiovascular events (adjusted HR 1.64, 95% CI 1.12–2.42), thus providing new evidence about the role of low-grade endotoxemia in favoring a pro-thrombotic phenotype in AF patients (6).

Wang et al. evaluated the safety and effectiveness of non-vitamin K antagonist oral anticoagulants (NOACs) compared to warfarin in AF patients with active, inactive and no peptic ulcer (PU) since patients with PU were excluded from the pivotal NOACs trials for stroke prevention because of the increased bleeding risk. Using a large cohort of patients from Taiwan who underwent esophagogastroduodenoscopy < 1 year before anticoagulation, the authors investigated the risks of major bleeding, gastrointestinal bleeding, and ischemic stroke/systemic embolism (IS/SE). In summary, 2,955 patients were included. In either patients with active or inactive PU, there were not significant differences in the risk of adverse outcomes between NOACs and warfarin users. However, in AF patients free of PU, NOACs were associated with significantly lower risks of major bleeding (aHR 0.26, 95% CI 0.12–0.53), gastrointestinal bleeding (aHR 0.25, 95% CI 0.01–0.59), and the composite of major bleeding, IS/SE, or death (aHR 0.64, 95% CI 0.46–0.89). It can be concluded that NOACs were as effective as warfarin in preventing IS/SE irrespective of PU status and safer than warfarin in reducing major bleeding in AF patients with endoscopic findings of no PU (7). This study highlights the value of screening for PU status before starting OAC in AF patients.

Liao et al., aimed to analyze and compare the incidence of ischemic bowel disease (IBD) in AF patients receiving warfarin or NOACs in a Taiwan nationwide cohort including newly diagnosed patients with AF aged ≥20 years without IBD from 2012 to 2018. In 69,549 AF patients under OAC, the annual incidence of IBD was 0.036%, whereas the highest incidence was observed in patients with CHA_2_DS_2_-VASc ≥2 (0.039% per year). The risk of IBD was similar between the NOACs and warfarin groups (aHR 0.80, 95% CI 0.50–1.34) during the follow-up, and even a propensity score matching analysis showed the same results (HR 0.88, 95% CI 0.42–1.77). In a subgroup analysis comparing each NOAC against warfarin, dabigatran offered a trend towards to lower risk of IBD (aHR 0.46, 95% CI 0.21–1.02, *p* = 0.057) (8). In summary, the annual incidence of IBD in AF patients taking OAC was low but presented a positive correlation with the increase of CHA_2_DS_2_-VASc score, and NOACs were comparable to warfarin in the risk of IBD.

In addition, Sánchez-Fuentes et al. performed a mini-review summarizing the current knowledge about drug-food/herbal products interactions of NOACs, and the potential implications of probiotics and prebiotics. In brief, this review demonstrated that there is still scarce evidence regarding this issue, but it should be considered that some herbals and food modulate P-gp and CYP3A4 activity hence their impact on the anticoagulant effect of NOACs cannot be neglected. On the other hand, novel therapies modifying gut microbiota composition and its activity such as probiotics and prebiotics might confer health benefits in users of NOACs, but the effect on the safety and efficacy on this family of anticoagulants is still unclear and warrants further research (9).

Finally, Cheng et al. presented a rare case of extensive submucosal esophageal hematoma developed after a Transesophageal echocardiography (TEE)-guided left atrial appendage occlusion (LAAO) procedure, in a 70-year-old man with persistent AF. Briefly, the patient reported persistent excruciating substernal chest pain and odynophagia after WATCHMAN implantation. A computed tomography scan revealed massive esophageal hematoma, which was confirmed later by Endoscopy and demonstrated esophageal mucosa exfoliation. Anticoagulation therapy was then discontinued and the authors adopted conservative management. After gradual symptomatic relief and a steady rise in the hemoglobin level, warfarin was reintroduced under close monitoring. This is a nice example of a rare complication of TEE-guided LAAO procedures. Some risk factors may increase the risk for esophageal hematoma, including abnormal hemostasis, inadequate manipulation of the TEE probe, and previous esophageal diseases. Hence, more data are required to determine the appropriate protocols for heparin dosing and monitoring during LAAO procedures, in order to prevent this complication. In addition, caution is recommended when selecting patients to undergo TEE-assisted procedures to avoid TEE-related esophageal hematoma, and a multidisciplinary approach is essential for devising the most effective strategies tailored to emergent cases (10).

This Research Topic clearly demonstrates that the management of AF should include a multidisciplinary approach, where different healthcare professionals provide their input on the appropriate care of AF patients. In this context, the interaction between the gastrointestinal system and AF requires particular attention and exploration.

## Author contributions

JR-C and GL drafted the manuscript. VR and FM made a critical revision of the manuscript. All authors contributed to the article and approved the submitted version.

## Conflict of interest

Author GL was consultant and speaker for BMS/Pfizer, Boehringer Ingelheim and Daiichi-Sankyo. Author JR-C was consultant for Idorsia Pharmaceuticals Ltd. The remaining authors declare that the research was conducted in the absence of any commercial or financial relationships that could be construed as a potential conflict of interest.

## Publisher's note

All claims expressed in this article are solely those of the authors and do not necessarily represent those of their affiliated organizations, or those of the publisher, the editors and the reviewers. Any product that may be evaluated in this article, or claim that may be made by its manufacturer, is not guaranteed or endorsed by the publisher.
